# Molecular Characterization of Mycolactone Producing Mycobacteria from Aquatic Environments in Buruli Ulcer Non-Endemic Areas in Côte d’Ivoire

**DOI:** 10.3390/ijerph14020178

**Published:** 2017-02-11

**Authors:** Marcellin B. Tano, Christelle Dassi, Lydia Mosi, Marina Koussémon, Bassirou Bonfoh

**Affiliations:** 1 Department of Food Sciences and Technology, Université Nangui Abrogoua, Abidjan, 02 BP 801 Abidjan 01, Côte d’Ivoire; tanobroumarcellin@yahoo.fr (M.B.T.); marinakoussemon@yahoo.fr (M.K.); 2 Department of Environment and Health, Centre Suisse de Recherches Scientifiques en Côte d’Ivoire (CSRS), Adiopodoumé, 01 BP 1303, Abidjan 01, Côte d’Ivoire; christelledassi@gmail.com (C.D.); bassirou.bonfoh@csrs.ci (B.B.); 3 UFR Biosciences, Université Félix Houphouët Boigny, Abidjan, 01 BP V 34 Abidjan 01, Côte d’Ivoire; 4 Department of Biochemistry, Cell and Molecular Biology, University of Ghana, Legon, P. O. Box LG 54, Legon, Ghana; 5 West African Centre for Cell Biology of Infectious Pathogens, University of Ghana, Legon, P. O. Box LG 54, Legon, Ghana

**Keywords:** non-tuberculous mycobacteria, mycolactone producing mycobacteria, environment, Buruli ulcer

## Abstract

Non-tuberculous mycobacteria (NTM), particularly mycolactone producing mycobacteria (MPM), are bacteria found in aquatic environments causing skin diseases in humans like Buruli ulcer (BU). Although the causative agent for BU, *Mycobacterium ulcerans* has been identified and associated with slow-moving water bodies, the real transmission route is still unknown. This study aimed to characterize MPMs from environmental aquatic samples collected in a BU non-endemic community, Adiopodoumé, in Côte d’Ivoire. Sixty samples were collected in four types of matrices (plant biofilms, water filtrate residues, plant detritus and soils) from three water bodies frequently used by the population. Using conventional polymerase chain reaction (PCR), MPMs were screened for the 16S ribosomal RNA (rRNA) mycobacterial gene, the IS*2404* insertion sequence, and MPM enoyl reductase (ER) gene. Variable Number Tandem Repeat (VNTR) typing with loci 6, 19, mycobacterial interspersed repetitive unit 1 (MIRU1) and sequence type 1(ST1) was performed to discriminate between different MPMs. Our findings showed 66.7%, 57.5% and 43.5% of positivity respectively for 16S rRNA, IS*2404* and ER. MPM discrimination using VNTR typing did not show any positivity and therefore did not allow precise MPM distinction. Nevertheless, the observed contamination of some water bodies in a BU non-endemic community by MPMs suggests the possibility of pathogen dissemination and transmission to humans. These aquatic environments could also serve as reservoirs that should be considered during control and prevention strategies.

## 1. Introduction

Non-tuberculous mycobacteria (NTM) are microorganisms which persist in the environment [1] and are notorious for causing serious opportunistic diseases, particularly in immunocompromised patients [2]. Mycolactone producing mycobacteria (MPM) are a sub-group of these NTMs which have been identified as pathogens responsible for infections both in humans and animals. This group comprises *Mycobacterium ulcerans*, rare select strains of *M. marinum*, *M. liflandii*, and *M. pseudoshotsii* [3].

Buruli ulcer (BU) is a neglected tropical disease endemic in Australia [4], South East Asia [5] and West Africa especially in Benin [6], Ghana [7] and Côte d’Ivoire [8], which is caused by *M. ulcerans*. The disease is very focal in its distribution in all reported countries, with defined endemic and non-endemic areas according to the reports from the various national diseases control centres [4,9,10]. In 2015, 549 new cases were reported in Côte d’Ivoire, which remains one of most affected countries [11,12]. *M. ulcerans* has been detected in slow moving water bodies in endemic regions [13,14]; however, its precise mode of transmission remains unclear. It is therefore important to assess the distribution of MPMs, particularly *M. ulcerans*, in both endemic and non-endemic communities in order to decipher the transmission mode to humans and identify the environmental reservoirs of these pathogens.

Adiopodoumé in Côte d’Ivoire is a non-endemic community where some cases of BU have been reported. The hypothesis that infection results from proximity to contaminated water bodies and socio-cultural practices of individuals along slow moving water bodies [13,15] has been proposed. The molecular detection of MPMs by polymerase chain reaction (PCR) amplification from environmental samples has been shown as essential for studying their ecology and transmission [10,16,17]. This is because several attempts to directly culture the mycobacterium from the environmental have been largely unsuccessful. Other studies have shown that aquatic plant biofilms, water filtrate residues, plant detritus and soil contain MPM DNA [10,18,19]. It has also been demonstrated that vegetables such as lettuce consumed by people could be contaminated by environmental mycobacteria [18]. The 16S ribosomal RNA (rRNA) mycobacterial gene and the insertion sequence (IS*2404*) present in *M. ulcerans* and other MPM species [10,20,21] have been amplified in diverse environmental samples collected from endemic water bodies. Currently, PCR identification of the IS*2404* insertion sequence is one of the World Health Organization (WHO) approved methods for the clinical diagnosis of BU in suspected patients [22]. Furthermore, a sequence encoding the enoyl reductase (ER) domain of MPM has been included in strain differentiation [10]. Additionally, variable number tandem repeat (VNTR) typing using the loci 6, 19, sequence type 1 (ST1) and mycobacterial interspersed repetitive unit 1 (MIRU1) has been also described as an extremely relevant method for distinguishing between MPMs [23].

In the present study, a systematic collection of 60 environmental samples from Adiopodoumé, a BU non-endemic area in Côte d’Ivoire, was undertaken as part of a global inter- and transdisciplinary research study which aimed to identify environmental sources of MPM (*M. ulcerans*) infection and risks associated with BU disease. Using PCR-based detection methods, MPMs were detected in three environmental water bodies suggesting their potential role as a source of contamination to the population in Adiopodoumé.

## 2. Materials and Methods

### 2.1. Study Site

Adiopodoumé is a village located in the southeast of Côte d’Ivoire. This village is surrounded by forest vegetation, several ponds, and the Ebrié lagoon. Its climate is subequatorial with an average ambient temperature of 26 °C and an annual precipitation of about 2000 mm. Adiopodoumé represents a non-endemic community where some suspected cases of BU (13 in 2009 [24]; 3 in 2014 (data from Adiopodoumé Health Centre) have been reported. The community environment has been modified with soil mining and the building of bridges. The population also uses the water bodies (lagoon, ponds) within this environment for their socio-economic activities like washing dishes, laundry, and gardening.

### 2.2. Aquatic Environmental Samples Collection

**Figure 1:**
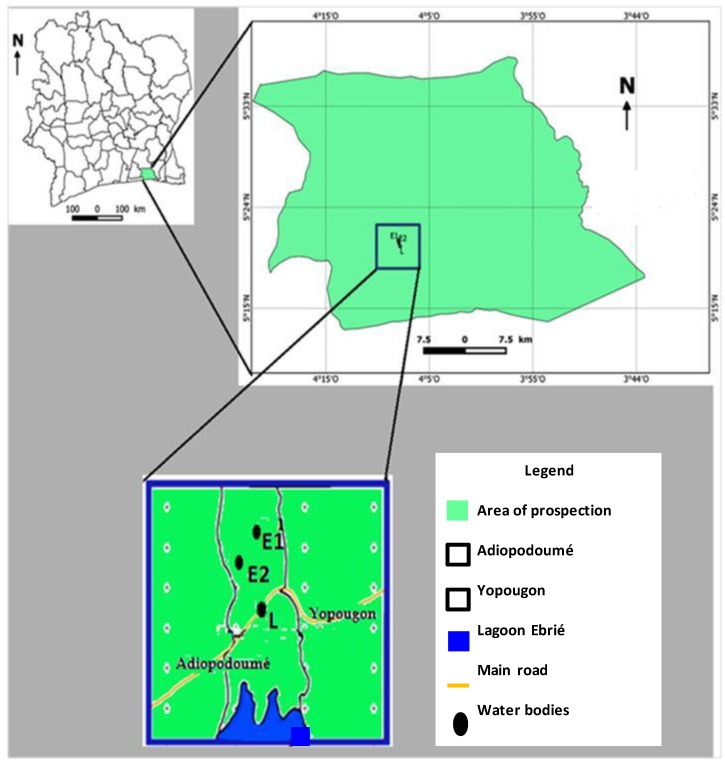
Study sampling site. Three water bodies were selected based on populations’ use and associated socio-cultural activities. L: Ebrié lagoon; E1: pond 1; E2: pond 2.

Sample collection was performed according to methods previously described by Narh et al. [21] with slight modifications. Three water bodies were selected (Figure 1) based on human use and associated socio-economic activities.

**Figure 2:**
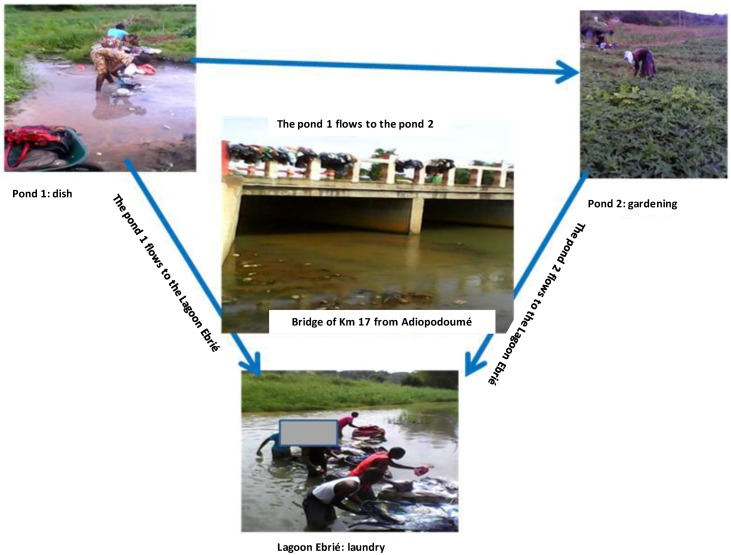
A proposed ecosystem of mycolactone producing mycobacteria in Adiopodoumé. Three water bodies (pond 1, pond 2 and Ebrié lagoon), frequently used by the community of Adiopodoumé for washing dishes, laundry, and gardening activities, were selected for the study. Pond 1 flowed into pond 2 and they both ran to Ebrié lagoon through the Adiopodoumé Km 17 Bridge. These environmental water bodies represent potential sources of infection by mycolactone producing mycobacteria (MPMs). The transmission of MPMs to humans could occur by direct contact with contaminated environmental samples through skin microtraumatism.

The proposed ecosystem of MPMs within Adiopodoumé community was subsequently described (Figure 2).

Sixty environmental samples were collected in four types of matrices (plant biofilms, water filtrate, plant detritus and soil) (Figure S1). Vegetable leaves (*Celosia argentea*, *Lactuca sativa*, *Xanthosoma esculenta*, *Basella alba*, *Ipomoea batatas*, *Hibiscus sabdariffa*, *Corchorus tridens* and *Abelmoschus esculentus*) as well as aquatic dominant plants were collected as plant samples. All samples were kept cool and transported to the laboratory for preservation at 4 °C until processing.

### 2.3. Molecular Characterization of Mycolactone Producing Mycobacteria

DNA extraction from environmental samples, as well as gel-based PCR reactions, were performed following the methods previously described by Williamson et al. and Narh et al. [10,21] with slight modifications. Negative controls (sterile water) were included for the DNA extraction. All gel-based PCR reactions were performed in an A200 gradient thermal cycler (LonGene, Hangzhou, China). All primers used are described in Table 1. Bovine Serum Albumin (40 ng, Inqaba Biotec, Pretoria, South Africa) was also added to environmental samples to relieve PCR inhibition in the amplification of all target loci. Negative (sterile water) and positive (*M. marinum* DL *240490* strain) controls were included for each run.

DNA extracted from environmental samples was initially screened for *Mycobacterium* spp. using mycobacteria-specific primers within the 16S rRNA gene. Briefly, the reaction was performed in a 25 μL reaction containing 1× PCR buffer (Thermo Scientific, Waltham, MA, USA), 2 mM MgCl_2_, 400 μM each of deoxyribonucleotide (Thermo Scientific), 160 nM each of forward and reverse primers (Inqaba Biotec), 1 U DreamTaq DNA polymerase (Thermo Scientific) and 5 μL of genomic DNA. The reaction was cycled at 95 °C for 3 min followed by 35 cycles each of denaturation at 95 °C for 45 s, annealing at 56 °C for 45 s and extension at 72 °C for 45 s. Final extension was at 72 °C for 10 min and reaction held at 4 °C. Seven microliters (7 uL) of PCR products were run on a 1.5% agarose gel (Thermo Scientific), stained with ethidium bromide (Thermo Scientific) and band sizes were estimated with 100 bp DNA ladder (Thermo Scientific).

Positive samples were then screened for the detection of non-tuberculous mycobacteria harboring the IS*2404* insertion sequence using an IS*2404* nested-PCR. Briefly, the first reaction was performed in a 25 μL reaction containing 1× PCR buffer (Thermo Scientific), 2 mM MgCl_2_, 300 μM each of deoxyribonucleotide (Thermo Scientific), 700 nM each of forward and reverse primers (Thermo Scientific), 1 U DreamTaq DNA polymerase (Thermo Scientific) and 5 μL of genomic DNA. The first reaction was cycled at 95 °C for 2 min followed by 40 cycles each of denaturation at 94 °C for 30 s, annealing at 66 °C for 45 s and extension at 72 °C for 1 min. Final extension was at 72 °C for 10 min and reaction held at 4 °C. The second reaction was performed with the same reagent concentrations as above, but 500 nM each of forward and reverse primers (Thermo Scientific) and 1 μL of PCR product from the first reaction. The second reaction was cycled at 95 °C for 2 min followed by 35 cycles each of, denaturation at 94 °C for 30 s, annealing at 67.7 °C for 45 s and extension at 72 °C for 45 s. Final extension was at 72 °C for 10 min and reaction held at 4 °C.

Samples which were IS*2404* positive were screened for the presence of the ER gene characterizing MPMs [10,21,23]. The ER-PCR reaction was also performed with the same reagent concentrations as described for the 16S rRNA PCR method and primers described in Table 1. The reaction was cycled at 95 °C for 2 min followed by 40 cycles each of denaturation at 94 °C for 1 min, annealing at 62.5 °C for 1 min and extension at 72 °C for 1 min. Final extension was at 72 °C for 10 min and reaction held at 4 °C.

MPM positive samples were processed for VNTR typing with four loci; locus 6, locus 19, MIRU1 and ST1 as described in previous studies [10,21,23] and with the same reagent concentrations as shown above. The reaction was cycled at 95 °C for 2 min followed by 40 cycles each of denaturation at 94 °C for 1 min, annealing at 58.5 °C for 1 min and extension at 72 °C for 1 min. Final extension was at 72 °C for 10 min and reaction held at 4 °C. In the reaction for ST1, the annealing was set at 63.1 °C for 1 min. 

Length polymorphism was estimated using PCR product sizes following separation on the agarose gel and recommendations by Williamson et al. and Narh et al. [10,21].

### 2.4. Statistical Analysis

Molecular data were recorded and stored in Microsoft Excel (Microsoft, Redmond, WA, USA). Statistical analysis of average proportions of matrices with positivity for mycobacteria markers was performed using R Version 3.1.2 (R Core Team, Vienna, Austria) and analysis of variance (ANOVA) with one factor and three repetitions. The comparison within matrix positivity was performed using the Fischer test and a *p value* < 0.05 was considered statistically significant.

## 3. Results

### 3.1. Distribution of Mycobacteria in Aquatic Environment from Adiopodoumé

The first screening for mycobacteria among the 60 environmental samples showed 40 (66.7%) positive samples for the gene encoding mycobacterial 16S rRNA with a 620 bp PCR product (Figure S2). Of the four different matrices, 12/18 plant biofilms (66.7%) were positive while all the 12 water filtrate residues were positive for this gene. Seven positive plant biofilms were from vegetable leaves (*Celosia argentea*, *Lactuca sativa*, *Xanthosoma esculenta*, *Basella alba*, *Ipomoea batatas*, *Hibiscus sabdariffa* and *Corchorus tridens*). Only 8/15 plant detritus and 8/15 soil samples (53.3%) were positive for the gene coding mycobacterial 16S rRNA (Table 2). Of the eight soil samples that tested positive, five were from the water edge and three were 5 m from the edge of the water body.

### 3.2. Distribution of Mycolactone Producing Mycobacteria in Aquatic Environment from Adiopodoumé Using IS2404 Typing

Samples that tested positive for mycobacterial DNA were then screened to identify mycolactone producing mycobacteria by the amplification of the IS*2404* insertion sequence. Twenty-three (57.5%) out of the previous 40 positive environmental samples were positive for IS*2404* insertion sequence with a PCR product size of about 210 bp (Figure S3). Among these positive samples, 8/12 (66.7%) were plant biofilms with four from vegetables leaves (*Celosia argentea, Lactuca sativa*, *Hibiscus sabdariffa* and *Corchorus tridens*), 6/12 (50%) water filtrate residues, 5/8 (62.5%) plant detritus and 4/8 (50%) soil samples (Table 3).

### 3.3. Distribution of Mycolactone Producing Mycobacteria in Aquatic Environment from Adiopodoumé Using ER Typing

Amplification of a 420 bp product on the macrolide-lincosamide-streptogramin A (MlsA) domain which encodes the ER domain of the pMUM001 plasmid of mycolactone producing mycobacteria resulted in only 10 positives out of the 23 IS*2404* positive environmental samples (Figure S4). Two of these ER-positive samples were aquatic plant biofilms and two water filtrate residues from Ebrié Lagoon. In addition, all the five detritus samples that tested positive for IS*2404* were also ER-positive. Only one soil sample from the water body edge was ER-positive (Table 4).

### 3.4. Discrimination between Mycolactone Producing Mycobacteria Species from the Adiopodoumé Aquatic Environment

The discrimination of ER positive samples using VNTR typing with locus 6, locus 19, MIRU1 and ST1 did not reveal any positive sample. Although VNTR typing was not successful in this, the results above confirm the presence of MPMs in environmental samples collected from Adiopodoumé water bodies.

## 4. Discussion

Buruli ulcer is a focal disease and in all affected countries, there are well-defined endemic and non-endemic communities. Cases found in non-endemic communities have been linked with a migration of affected persons from endemic areas [4]. Although the mode of transmission is still unknown, the causative agent, *M. ulcerans* has been detected in water bodies of endemic communities. The present study focused on the detection of MPM, particularly *M. ulcerans* from environmental samples collected from aquatic environments in a BU non-endemic community.

The results showed the presence of mycobacteria in 40 (66.7%) samples after PCR amplification of the gene encoding 16S rRNA. Stinear et al. [17] showed the usefulness of 16S rRNA by asserting that this gene would distinguish between *M. ulcerans* to *M. marinum*. This study showed that 100% of water samples tested were positive for the gene coding the 16S rRNA gene of mycobacteria. This implies that water is the major source of these organisms as reported by Williamson et al. and Narh et al. [10,21] in Ghana with plant biofilms, water, detritus and soils contaminated by mycobacteria. Other authors like Brou et al. [14] in Côte d’Ivoire and Aiga et al. [13] in Ghana, have shown that risk factors for BU were linked with water.

This result also suggests that mycobacteria are found in the samples from the environment. The presence of these mycobacteria in Adiopodoumé environment could be explained by the fact that in this community, there are many stagnant water bodies which receive inflows from other slow moving water possibly transporting the pathogen. Some authors as Raghunathan et al. [15] reported that in Ghana slow moving streams were the main sources of mycobacteria. Adiopodoumé is also located in an urbanized area. In a report by Radomski [26], urban areas were found to be affected by mycobacterial species due to the proximity of surface water.

We also included another PCR method based on the amplification of the IS*2404* insertion sequence. This sequence has been used for the detection of *M. ulcerans* and other MPMs in environmental samples because of the high copy number of the insertion sequence (IS) element (213 copies) within their plasmid genome [17]. The amplification of the IS*2404* insertion sequence in the present study revealed that 23 samples were positive for this marker. This result highlights the presence of MPMs in environmental samples collected from Adiopodoumé water bodies and it is supported by previous studies in Ghana and South Australia [10,17,21,27]. Furthermore, in Côte d’Ivoire, N’Gazoa-Kacou and her team also assessed *M. ulcerans* DNA presence by IS*2404* real-time PCR in clinical and environmental samples team [28]. Stinear et al. conferred that IS*2404*-PCR is a preliminary positive test for the identification of *M. ulcerans* in environmental samples [29].

The present study also highlights the presence of MPMs using IS2*404* insertion sequence detection in vegetable samples *Lactuca sativa*, *Celosia argentea*, *Hibiscus sabdariffa* and *Corchorus tridens*. These results are supported by those of Marsollier et al. [30] who showed biofilms formed by *M. ulcerans* on aquatics plants and an IS*2404* positive plant (Scrophulariaceae family) in Côte d’Ivoire.

We went on to further detect the presence of the ER gene encoding a polyketide synthase domain of the mycolactone producing plasmid pMUM001 [10]. The samples tested positive for the IS*2404* insertion sequence were processed for ER-PCR amplification as a confirmatory test for the identification of *M. ulcerans* and other MPMs (*M. marinum DL*, *M. liflandii*, and *M pseudoshottsii*) [21,29]. Our data showed that from the 23 IS*2404* positive samples, 10 (43.5%) tested positive after ER-PCR, confirming that aquatic environments in Adiopodoumé were contaminated by MPMs. These results are supported by those of Williamson et al. and Narh et al. [10,21] in Ghana, where MPMs were detected in environmental samples from BU non-endemic and endemic areas by ER-PCR. 

Our attempts to further discriminate MPMs by amplification of VNTR loci were not successful in any of the ER-positive environmental samples tested. That may be explained by a low quantity of DNA, from tested environmental samples, not sufficient for amplifying these markers and then typing MPMs. An inverse result was found by Williamson et al. and Narh et al. [10,21] in their studies in Ghana where the same VNTR loci were successfully amplified in environmental samples. Thus, this experiment needs to be repeated under different conditions, using a high quantity of DNA for PCR with confirmation by DNA sequencing and/or more sensitive amplification methods like quantitative PCR, in order to identify more precisely the MPMs involved in aquatic environment contamination in Adiopodoumé community.

## 5. Conclusions

The screening of mycolactone producing mycobacteria, using gel-based PCR in environmental samples has shown 66.7% of positivity for 16S rRNA mycobacterial gene in tested samples. From these positive samples, 57.5% were also positive for the IS*2404* insertion sequence and 43.5% showed positivity for the enoyl reductase gene. Thus, the present study underscores the distribution of MPMs in samples collected within an aquatic environment from Adiopodoumé, a historically non-endemic community for Buruli ulcer disease but where some cases were reported these last years. This distribution was homogeneous among the different types of samples collected in this environment.

Furthermore, VNTR loci typing of these samples did not reveal any positivity that allows the discrimination between *M. ulcerans* and other MPMs. Nevertheless, these preliminary results showed that more attention should be placed on aquatic environments from BU non-endemic area and environmental samples from there which could be sources of mycobacteria infection in humans using these water bodies and living in these areas.

## Figures and Tables

**Table 1:** Specific primers used for identification of non-tuberculous mycobacteria.

Primer Name	Forward and Reverse Primer Sequences	Size of Amplicon (bp)	References
**16S rRNA**	PA: 5′-AGAGTTTGATCCTGGCTCAG-3′ MSHA: 5′-AAAAAGCGACAAACCTACGAG-3′	620	[25]
**IS*2404* Nested 1**	pGp1: 5′-AGGGCAGCGCGGTGATACGG-3′ pGp2: 5′-CAGTGGATTGGTGCCGATCGAG-3′	400	[5]
**IS*2404* Nested 2**	pGp3: 5′-GGCGCAGATCAACTTCGCGGT-3′ pGp4: 5′-CTGCGTGGTGCTTTACGCGC-3′	210	[5]
**ER**	LMF: 5′-GAGATCGGTCCCGACGTCTAC-3′ LMR: 5′-GGCTTGACTCATGTCACGTAAG-3′	420	[10]
**Locus 6**	R-5′-GACATCGAAGAGGTGTGCCGTCT-3′ F-5′-GACCGTCATGTCGTTCGATCCTAGT-3′	variable	[10]
**Locus 19**	R-5′-TGGCGACGATCGAGTCTC-3′ F-5′-CCGACGGATGAATCTGTAGGT-3′	variable	[10]
**MIRU1**	R-5′-GCCCTCGGGAATGTGGTT-3′ F-5′-GCTGGTTCATGCGTGGAAG-3′	variable	[10]
**ST1**	R-5′-CGCCACCCGCGGACACAGTCG-3′ F-5′-CTGAGGGGATTTCACGACCAG-3′	variable	[10]

ER: enoyl reductase; MIRU1: mycobacterial interspersed repetitive unit 1; rRNA: ribosomal RNA; ST1: sequence type 1.

**Table 2:** Distribution of mycobacteria in aquatic environmental matrices from Adiopodoumé.

Environmental Samples	16S rRNA Positives (%)
Plant biofilms	12/18 (66.7 ^a^)
Water filtrates	12/12 (100 ^a^)
Plant detritus	8/15 (53.3 ^a^)
Soils	8/15 (53.3 ^a^)

^a^ There was no significant difference between the positive average proportions of the four types of environmental matrices collected and the distribution of mycobacteria in environmental samples could be homogeneous; *p* value (0.41) > 0.05 for 16S rRNA.

**Table 3:** Distribution of IS*2404* positive mycolactone producing mycobacteria in aquatic environmental matrices from Adiopodoumé.

Environmental Samples	IS*2404* Positives (%)
Plant biofilms	8/12 (66.7 ^a^)
Water filtrates	6/12 (50 ^a^)
Plant detritus	5/8 (62.5 ^a^)
Soils	4/8 (50 ^a^)

^a^ There was no significant difference between the positive average proportions of the four types of environmental matrices collected and the distribution of mycolactone producing mycobacteria in environmental samples could be homogeneous; *p*-value (0.72) > 0.05 for IS*2404*.

**Table 4:** Distribution of ER-positive mycolactone producing mycobacteria in aquatic environmental matrices from Adiopodoumé.

Environmental Samples	ER-Positives (%)
Plant biofilms	2/8 (25 ^a^)
Water filtrates	2/6 (33.3 ^a^)
Plant detritus	5/5 (100 ^a^)
Soils	1/4 (25 ^a^)

^a^ There was no significant difference between the positive average proportions of the four types of environmental matrices collected and the distribution of mycolactone producing mycobacteria in environmental samples could be homogeneous; *p*-value (0.14) > 0.05 for ER.

## Supplementary Materials

The following are available online at http://www.mdpi.com/1660-4601/14/2/178/s1, Figure S1: Aquatic environmental sample collection, Figure S2: Polymerase chain reaction profile obtained after amplification of the 16S ribosomal RNA (rRNA) gene of mycobacteria; Figure S3: Polymerase chain reaction profile obtained after amplification of the IS*2404* insertion sequence in non-tuberculous mycobacteria, Figure S4: Polymerase chain reaction profile obtained after amplification of the enoyl reductase gene of mycolactone producing mycobacteria.

## References

[1] Kazda J. (2009). The Chronology of Mycobacteria and the Development of Mycobacterial Ecology. The Ecology of Mycobacteria.

[2] N’Guessan K., Kouassi Y., Bouzid S., Ehuie P., Koffi K., Oniangue C., Aka N., Dosso M. (2001). Value and limits of microscopy of exudates in *Mycobacterium ulcerans* cutaneous infection in Côte d’Ivoire. Bull. Soc. Pathol. Exot..

[3] Ranger B.S., Mahrous E.A., Mosi L., Adusumilli S., Lee R.E., Colorni A., Rhodes M., Small P.L. (2006). Globally distributed mycobacterial fish pathogens produce a novel plasmid-encoded toxic macrolide, mycolactone F. Infect. Immun..

[4] Merritt R.W., Walker E.D., Small P.L., Wallace J.R., Johnson P.D., Benbow M.E., Boakye D.A. (2010). Ecology and transmission of Buruli ulcer disease: A systematic review. PLoS Negl. Trop. Dis..

[5] Ablordey A., Amissah D.A., Aboagye I.F., Hatano B., Yamazaki T., Sata T., Ishikawa K., Katano H. (2012). Detection of *Mycobacterium ulcerans* by the loop-mediated isothermal amplification method. PLoS Negl. Trop. Dis..

[6] Debacker M., Aguiar J., Steunou C., Zinsou C., Meyers W.M., Guédénon A., Scott J.T., Dramaix M., Portaels F. (2004). *Mycobacterium ulcerans* Disease (Buruli Ulcer) in Rural Hospital, Southern Benin, 1997–2001. Emerg. Infect. Dis..

[7] Amofah G., Bonsu F., Tetteh C., Okrah J., Asamoa K., Asiedu K., Addy J. (2002). Buruli ulcer in Ghana: Results of a national case search. Emerg. Infect. Dis..

[8] Ahoua L., Aka N., Ekaza E., Bouzid S., N’Guessan R., Dosso M. (2009). Risk factors for Buruli ulcer in Côte d’Ivoire: Results of a case-control study. Afr. J. Biotechnol..

[9] Wagner T., Benbow M.E., Brenden T.O., Qi J., Johnson R.C. (2008). Buruli ulcer disease prevalence in Benin, West Africa: Associations with land use/cover and the identification of disease clusters. Int. J. Health Geogr..

[10] Williamson H.R., Benbow M.E., Nguyen K.D., Beachboard D.C., Kimbirauskas R.K., McIntosh M.D., Quaye C., Ampadu E.O., Boakye D., Merritt R.W. (2008). Distribution of *Mycobacterium ulcerans* in Buruli ulcer endemic and non-endemic aquatic sites in Ghana. PLoS Negl. Trop. Dis..

[11] Kanga J.M., Kacou D.E. (2001). Aspects épidémiologiques de l’ulcère de Buruli en Côte d’Ivoire: Résultats d’une enquête nationale. Bull. Soc. Pathol. Exot..

[12] (2014). Résumé du bilan 2013 du Programme National de Lutte contre l'ulcère de Buruli. Aspect clinique, épidémiologique et thérapeutique.

[13] Aiga H., Amano T., Cairncross S., Adomako J., Nanas O.K., Coleman S. (2004). Assessing water-related risk factors for Buruli ulcer: A case-control study in Ghana. Am. J. Trop. Med. Hyg..

[14] Brou T., Broutin H., Elguero E., Asse H., Guegan J.F. (2008). Landscape diversity related to Buruli ulcer disease in Côte d’Ivoire. PLoS Negl. Trop. Dis..

[15] Raghunathan P.L., Whitney E.A., Asamoa K., Stienstra Y., Taylor T.H., Amofah G.K., Ofori-Adjei D., Dobos K., Guarner J., Martin S. (2005). Risk factors for Buruli ulcer disease (*Mycobacterium ulcerans* Infection): Results from a case-control study in Ghana. Clin. Infect. Dis..

[16] Ekaza E., Kacou-N’douba A., Oniangue N.C., Ehuie P., N’Guessan K.R., Aka N., Bouzid S.A., Faye-Kette H., Dosso M. (2004). Apport de l’amplification génique dans la détection de *Mycobacterium ulcerans* dans les exsudats et les biopsies cutanées en Côte d’Ivoire. Bull. Soc. Pathol. Exot..

[17] Stinear T.P., Seemann T., Pidot S., Frigui W., Reysset G., Garnier T., Meurice G., Simon D., Bouchier C., Ma L. (2007). Reductive evolution and niche adaptation inferred from the genome of *Mycobacterium ulcerans*, the causative agent of Buruli ulcer. Genome Res..

[18] Yoder S., Argueta C., Holtzman A., Aronson T., Berlin O.G.W., Tomasek P., Glover N., Froman S., Stelma G.J. (1999). PCR comparison of *Mycobacterium avium* isolates obtained from patients and foods. Appl. Environ. Microbiol..

[19] Lavender C.J., Stinear T.P., Johnson P.D., Azuolas J., Benbow M.E., Wallace J.R., Fyfe J.A. (2008). Evaluation of VNTR typing for the identification of *Mycobacterium ulcerans* in environmental samples from Victoria, Australia. FEMS Microbiol. Lett..

[20] Fyfe J.A., Lavender C.J., Johnson P.D.R., Globan M., Sievers A., Azuolas J., Stinear T.P. (2007). Development and application of two multiplex real-time PCR assays for the detection of *Mycobacterium ulcerans* in clinical and environmental samples. Appl. Environ. Microbiol..

[21] Narh C.A., Mosi L., Quaye C., Dassi C., Konan D.O., Tay S.C.K., de Souza D.K., Boakye D.A., Bonfoh B. (2015). Source Tracking *Mycobacterium ulcerans* Infections in the Ashanti Region, Ghana. PLoS Negl. Trop. Dis..

[22] Portaels F., World Health Organization (2014). Laboratory Diagnosis of Buruli Ulcer: A Manual for Health Care Providers.

[23] Williamson H., Phillips R., Sarfo S., Wansbrough-Jones M., Small P. (2014). Genetic Diversity of PCR-Positive, Culture-Negative and Culture-Positive *Mycobacterium ulcerans* Isolated from Buruli Ulcer Patients in Ghana. PLoS ONE.

[24] Aka N., Ekaza E., Coulibaly-Ngolo M.D., Kouadio K., Koffi L., Coulibaly B., Kodia M., Ngazoa-Kakou S., N’Guessan K.R., Yapo-Crézoit A. (2010). Buruli ulcer in Côte d’Ivoire: a new disease focus identified in Adiopodoumé, Yopougon commune (Abidjan, Km 17). Abstracts of the annual meeting of the WHO Global Ulcer Initiative, 22–24 March 2010.

[25] Hughes M.S., Skuce R.A., Beck L.A., Neill S.D. (1993). Identification of mycobacteria from animals by restriction enzyme analysis and direct DNA cycle sequencing of polymerase chain reaction-amplified 16S rRNA gene sequences. J. Clin. Microbiol..

[26] Radomski N. (2011). Sources des mycobactéries non tuberculeuses dans les bassins versants. Ph.D. Thesis.

[27] Kirschner P., Bottger E.C., Parish T.S.N. (1998). Species identification of mycobacteria using rDNA sequencing. Mycobacteria Protocols (Methods in Molecular Biology).

[28] Ngazoa-Kakou E.S., Ekaza E., Aka N., Coulibaly-N’Golo D., Coulibaly B., Dosso M. (2011). Evaluation of real-time PCR for *Mycobacterium ulcerans* in endemic region in Côte d’Ivoire. Afr. J. Microbiol. Res..

[29] Stinear T.P., Pryor M.J., Porter J.L., Cole S.T. (2005). Functional analysis and annotation of the virulence plasmid *pMUM001* from *Mycobacterium ulcerans*. Microbiology.

[30] Marsollier L., Stinear T., Aubry J., Saint André J.P., Robert R., Legras P., Manceau A.-L., Audrain C., Bourdon S., Kouakou H. (2004). Aquatic Plants Stimulate the Growth of and Biofilm Formation by *Mycobacterium ulcerans* in Axenic Culture and Harbor These Bacteria in the Environment. Appl. Environ. Microbiol..

