# Supplementary material for: Molecular Characterization of Mycolactone Producing Mycobacteria from Aquatic Environments in Buruli Ulcer Non-Endemic Areas in Côte d’Ivoire
Source: Int J Environ Res Public Health. 2017 Feb 11;14(2):178. doi: 10.3390/ijerph14020178 (PMC5334732; doi:10.3390/ijerph14020178)
Supplement: Supplementary file 1 [file ijerph-14-00178-s001.pdf]

Supplementary Materials: Molecular Characterization of Mycolactone Producing Mycobacteria from Aquatic Environments in Buruli Ulcer Non-Endemic Areas in Côte d'Ivoire

Marcellin B. Tano, Christelle Dassi, Lydia Mosi, Marina Koussémon and Bassirou Bonfoh

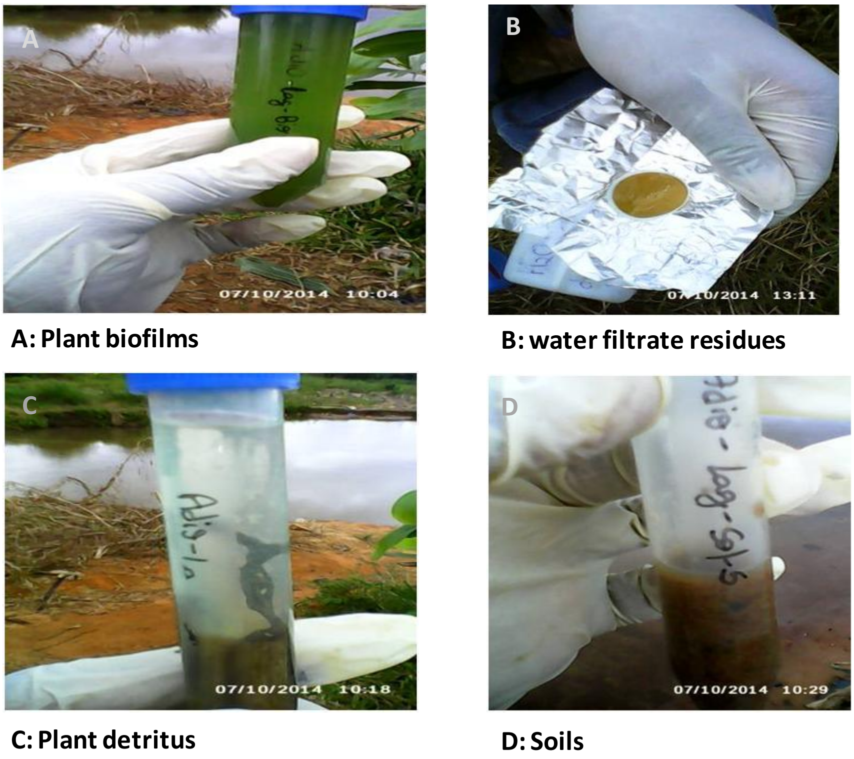

Figure S1. Aquatic environmental sample collection**.** Environmental matrices were collected from each of the three water bodies and comprised four types of matrices: (**A**) plant biofilms, (**B**) water filtrate residues, (**C**) plant detritus and (**D**) soils.

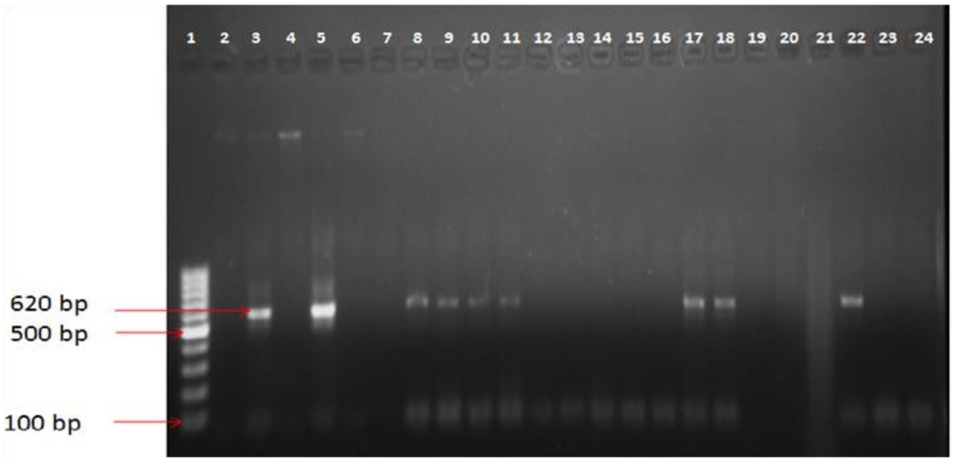

Figure S2. Polymerase chain reaction profile obtained after amplification of the 16S ribosomal RNA (rRNA) gene of mycobacteria. Lane 1: 100 bp DNA ladder; lanes 2–7: plant biofilms; lanes 8–11: water filtrate residues; lanes 12–16: plant detritus; lanes 17–21: soil; lane 22: positive control; lanes 23 and 24: negative controls.

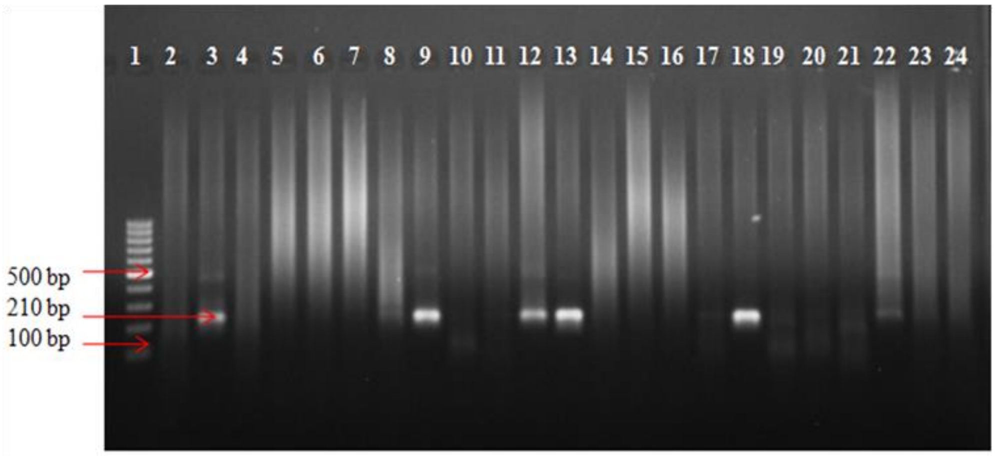

Figure S3. Polymerase chain reaction profile obtained after amplification of the IS*2404* insertion sequence in non-tuberculous mycobacteria. Lane 1: 100 bp DNA ladder; lanes 2, 3 and 12–15: water filtrate residues; lanes 4, 5 and 21: soil; lanes 6–11: plant biofilms; lanes 16–20: plant detritus; lane 22: positive control; lanes 23 and 24: negative controls.

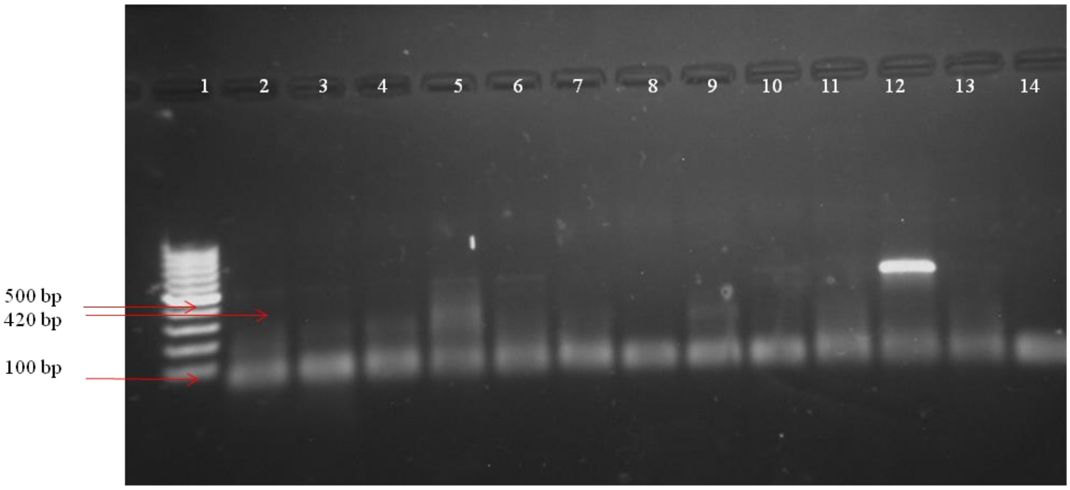

Figure S4. Polymerase chain reaction profile obtained after amplification of the enoyl reductase gene of mycolactone producing mycobacteria. Lane 1: 100 bp DNA ladder; lanes 2–5 and 11: plant detritus; lanes 6 and 7: plant biofilms and vegetable; lanes 8 and 9: water filtrate residues lane 10: soil; lane 12: positive control; lanes 13 and 14: negative controls.
